# METABOLOMIC SIGNATURES PREDICT HEALTHY AGING IN COMMUNITY-DWELLING OLDER ADULTS

**DOI:** 10.1093/geroni/igad104.2771

**Published:** 2023-12-21

**Authors:** Shanshan Yao, Megan Marron, Robert Boudreau, Anne Newman, Ravi Shah, Venkatesh Murthy

**Affiliations:** University of Pittsburgh School of Public Health, Pittsburgh, Pennsylvania, United States; University of Pittsburgh School of Public Health, Pittsburgh, Pennsylvania, United States; University of Pittsburgh School of Public Health, Pittsburgh, Pennsylvania, United States; University of Pittsburgh School of Public Health, Pittsburgh, Pennsylvania, United States; Vanderbilt University Medical Center, Nashville, Tennessee, United States; University of Michigan, Ann Arbor, Michigan, United States

## Abstract

This study sought to identify metabolomic signatures of multi-system aging by relating comprehensive mass-spectrometry-based metabolomic profiles with the aging phenome. We studied 2469 Black and White older adults (mean age, 75 years; 38% Black) in the Health, Aging, and Body Composition Study (Health ABC) with detailed characterization of the aging phenome across 20 aging-related phenotypes spanning 7 domains (physical function, neurocognitive/executive function, adiposity/sarcopenia, vascular aging, dietary pattern, psychosocial stress, and physical activity). Regression was used to identify metabolites associated with the aging phenome for pathway analytics. Penalized regression and principal components analysis were used to generate parsimonious, metabolomic scores, which were entered into Cox models for the endpoint of exceptional healthy aging, defined as surviving free of cardiovascular disease, cancer, dementia, or major disability over 9 years. We identified 4 distinct metabolomic scores independently linked to body composition, physical performance/mental health, muscle strength, and physical activity. The body composition score (hazard ratio (HR)=0.80 per SD [95% Confidence Interval: 0.70–0.90]), mental/physical performance score (0.77 [0.69–0.87]), and muscle strength score (0.82 [0.73–0.93]) were associated with failure to achieve “exceptionally healthy aging” after adjusting for age, race, gender, and lifestyle and traditional risk factors. Metabolomic signatures of different aspects of the aging phenome are associated with long-term healthy aging processes independent of lifestyle and traditional risk factors. Efforts to include precision measures of metabolomic health in risk stratification to achieve healthy aging are warranted. External validation in a diverse cohort of younger adults is underway.

